# The trend and pattern of adult mortality in South-Central Ethiopia: analysis using the 2008-2019 data from Butajira Health and Demographic Surveillance System

**DOI:** 10.1080/16549716.2022.2118180

**Published:** 2022-09-30

**Authors:** Hailelule Aleme Yizengaw, Wubegzier Mekonnen Ayele, Alemayehu Worku Yalew

**Affiliations:** School of Public Health, College of Health Sciences, Addis Ababa University, Addis Ababa, Ethiopia

**Keywords:** Adult mortality, burden, HDSS, pattern, trend

## Abstract

**Background:**

Understanding context-specific temporal trends in mortality is essential for setting health policy priorities.

**Objective:**

To investigate the trends and distribution of deaths due to communicable and non-communicable diseases and external causes in South-Central Ethiopia.

**Method:**

All adult deaths captured by the Butajira Health and Demographic Surveillance System between January 2008 and December 2019 were included. A verbal autopsy method of collecting cause of death data was used. Physician review and a computerised algorithm, InterVA, were used to determine the cause of death. Coding was undertaken using the World Health Organization's International Classification of Diseases. Trends in adult mortality rate and proportional mortality were estimated by major cause of death categories. Significant trends were analysed using the Mann–Kendall statistical test with a significance set at *P* < 0.05. Deaths were also disaggregated by age, sex, and residence.

**Results:**

There were 1,612 deaths in 279,681 person-years; 811 (50.3%) were females. The median age at death was 65 years. The proportional adult mortality and adult mortality rates (per 1000 person-years) attributed to communicable diseases, non-communicable diseases, and external causes were 31.1%, 58.9%, and 6.0%, and 1.9, 3.4, and 0.4, respectively. Adult mortality due to communicable diseases showed a declining trend (*tau*, the measure of the strength and direction of association, = −0.52; *P* < 0.05), whereas the trend increased for non-communicable diseases (*tau* = 0.67, *P* < 0.05) and external causes (*tau* = 0.29, *P* > 0.05). Moreover, death rates were pronounced in the 65+ age group and rural areas but comparable among males and females.

**Conclusion:**

The trend in deaths due to communicable diseases declined but increased for non-communicable diseases and external causes with significant public health burdens. These findings will provide essential input in formulating health policy reforms to reduce premature mortality.

## Background

Reliable and timely mortality and cause of death (CoD) data show the levels, patterns, and trends in deaths [1]. These data are critically important and provide essential policy inputs for well-informed decision-making and public health interventions to avert preventable mortality [1,2].

Mortality and CoD data are collected through various methods [3]. High-income countries generally have robust vital events registration systems, whereas obtaining reliable data is a pervasive challenge for many developing countries [4]. Due to this discrepancy, each year, virtually two-thirds of the world’s deaths do not have a reliably recorded cause [5], especially in an area where deaths occur at home [6] and mortality is highest [7–9].

Although not without limitations, verbal autopsy (VA) can provide useful information on population CoD [9]. The Health and Demographic Surveillance System (HDSS) sites are the primary source of community-based VA data [10]. In the HDSS, verbal autopsy (VA) data are routinely collected through cyclical visits to the household on the systematically identified and registered death.

For more than 25 years, VA has been a well-functioning surveillance method for over 45 low- and middle-income countries (LMICs) [11], and its application can potentially influence policy outside of research contexts [12]. Various VA-based studies done in LMICs show the burden, trends, and patterns of death overtime [12]. Pragmatically, ample experience with the HDSS in VA can be an interim solution to fill the gaps in CoD identification activities in the civil registration and vital statistics system [13,14]. Recently, significant efforts have been employed to ensure the analytical potential of VA [9], and there have been methodological advances in applying VA to determine the CoD [15]. So far, there have been different VA interpretation methods [16]; traditionally, the underlying CoD has been determined by a physician [17]. However, since 2000, a Bayes’ Theorem-based, expert-driven probabilistic model (like the InterVA4) has been used for interpreting VA data [17,18].

Globally, communicable diseases (CDs) have been the main CoD for centuries. Although the global burden is decreasing [19], CDs remain a major public health threat, specifically in emerging or re-emerging situations [20,21]. The current COVID-19 pandemic and other CD outbreaks demonstrate gaps in CD control [22]. Non-communicable diseases (NCDs) are becoming the leading CoD and increasing global concerns [23]. The World Health Organization (WHO) 2018 report indicated that 71% of the global deaths are attributed to NCDs, 78% of which occur in LMICs [24]. This highlights the important changes in population health [25]. According to one study conducted in 2014 across 21 International Network of Demographic Evaluation of Population and Their Health (INDEPTH) sites, NCDs made a significant contribution to CoD [26]. Moreover, deaths and disabilities associated with external causes (ECs) are a significantly important CoD and add to the global health burden [25]. Ethiopia is no exception [27].

Over 90% of deaths in Ethiopia occur at home. Many deaths are not registered, and information on CoD is rarely recorded [28]. This paucity of information critically impedes understanding the burden and trends in the CoD due to diseases and ECs, particularly in rural settings. This study, therefore, aims to assess the adult mortality pattern and trends in CDs, NCDs, and ECs in south-central Ethiopia using the Butajira Health and Demographic Surveillance System (HDSS) database.

## Methods

### Study setting, population, and design

Butajira HDSS is in south-central Ethiopia within the Gurage Zone of Southern Nations, Nationalities, and People’s Region (SNNPR), approximately 130 km south of Addis Ababa, the capital city of Ethiopia. The Butajira HDSS was initiated in 1987 following a population census of nine randomly selected peasant associations in rural villages or Kebeles (the smallest administrative unit) and one urban dwellers’ association (urban village) selected from 82 rural and 4 urban Kebeles using the probability proportional to size technique [27,29]. This study used a longitudinal population-based surveillance design. All residents of the Butajira HDSS were included. Mortalities captured between 1 January 2008 and 31 December 2019 were obtained from the electronic database maintained by the Butajira HDSS. Adult deaths (15 years and above) were retrieved. Age at death was classified as young adults (15–49 years), middle-aged adults (50–64 years), and old-aged adults (65+ years) [30].

### Death event capturing and the cause of death surveillance system

Butajira HDSS has two teams (demographic data collection team and CoD data collection team) with data collectors and supervisors. Standard INDEPTH-Network event registration forms were routinely used to record births, deaths, changes in marital and immigrant status, and the formation of new households. Village-based data were collected manually on paper. Each Kebele was divided into eight equal parts to ease monitoring and the periodicity of capturing vital events. The events happening in one-eighth of a Kebele were recorded from Monday through Thursday each week. On Friday, the completed forms were transferred to the project office for data entry.

Deaths captured by the demographic data collection team were shared with the CoD data collection team to conduct VA after the cultural mourning period. The VA involves a structured interview with the next of kin or a caregiver about signs, symptoms, and events the deceased experienced before death [30]. Since 2007, VA procedures have been conducted on all deaths in the Butajira HDSS. Signs, symptoms, and events leading to death were captured using standard forms prepared for neonates (age at death 0–28 days), children (29 days–14 years), and adults (15 years plus). The data collection tools had two major parts: pre-coded questions and a place where one can write narratives of signs, symptoms, and events leading to death as well as past illness history, duration and frequency of illness, severity and continuity, location, associated systems, and medical history. Until May 2017, physicians coded VA data, but thereafter, the InterVA4 probabilistic method has been used.

### Physician-certified VA method (PCVA)

The PCVA method was employed to determine the CoD for data recorded from January 2008 to April 2017. Two trained and blinded physicians independently reviewed the completed VA questionnaires and assigned codes and titles for each CoD as underlying, immediate, and contributing factors using the World Health Organization’s International Classification of Diseases Version 10 (ICD-10) manual. If there was any disagreement, the case was sent to a third physician. If two of these three physicians agreed, the final CoD was assigned. If not, the case was labelled as undetermined. CoD was assigned as ‘unspecified CoD (VA-99)’ in a situation where no specific CoD was identified.

### The InterVA/computer-coded VA method (CCVA)

InterVA uses probabilistic modelling to arrive at a likely CoD for each VA case compatible with the ICD-10 [26,31]. The InterVA-4 model assigns one to three likely causes and statistical likelihoods. If the sum of their likelihoods was less than 1, then the residual component was assigned as indeterminate. In a minority of cases, for example, where symptoms were vague, contradictory, or mutually inconsistent, or InterVA-4 could not determine a CoD, these deaths were attributed as ‘indeterminate’ [26,31]. The InterVA4 model version 4.02 was used to determine the CoD for the dataset collected between May 2017 and December 2019. Finally, the interVA4 CoD codes were translated into ICD-10 equivalent codes. There are no significant differences between physician interpretation and the InterVA4 modelling since broad categories of CoD were used [32]. Finally, the two datasets (with PCVA and CCVA) were combined into similar CoD categories and analysed (details available in Supplementary Table 1).

### Classification of causes of death

The burden of diseases and conditions worldwide is grouped under three important categories, CDs, NCDs, and ECs [33], that cause significant deaths and disabilities [24,33,34]. ICD-10 was used to classify the CoD. Diseases like sepsis, acute respiratory infection (including pneumonia), HIV/AIDS-related deaths, pulmonary tuberculosis, diarrheal diseases, malaria, measles, meningitis, encephalitis, tetanus, and other disease conditions of a similar kind were included under CDs. The NCDs group included disorders such as neoplasms, cardiovascular disorders, gastrointestinal disorders, renal disorders, respiratory disorders, mental and behavioural disorders, and other related conditions. Road traffic accidents, accidental falls, accidental drowning and submersion, accidental exposure to smoke, fire, or flames, contact with venomous animals and plants, intentional self-harm, assault, and exposure to forces of nature were assigned to ECs or injuries.

### Data quality assurance

The quality assurance mechanism put in place for the longitudinal data has been detailed elsewhere [35,36].

### Data management and analysis

For all adult deaths, the socio-demographic data from the HDSS database and CoD from the verbal autopsy database were linked using a unique individual ID. After editing and coding the data, cases were categorised into broad CoD, including CDs, NCDs, and ECs. Then, annualised crude and stratified broad cause-specific mortality rates and proportional mortality were calculated. Changes in the adult mortality rate (per 1,000 person-years) and proportional mortality in the broad CoD category were the main indicators used to assess adult mortality patterns and trends. The annual mortality levels were first checked for serial correlation using autocorrelation and partial autocorrelation plots [37]. In addition, the Mann–Kendall statistical test was used to detect a trend in the data. A positive value of *tau* (Kendall’s tau statistic) is an indicator of an increasing trend, whereas its negative value is indicative of a decreasing trend [38]. A significant trend was assumed where *P* < 0.05 using the Z statistic [37,39]. Data editing and the computing of descriptive summaries were managed in Microsoft Excel worksheets. Further analyses were carried out using the R-Statistical software package version 3.6.3.

## Results

### Socio-demographic characteristics of the deceased

During the study period (1 January 2008 to 31 December 2019), a total of 1,612 deaths were captured in 279,681 person-years. The CCVA and PCVA methods were assigned CoD for 99% and 96% of cases, respectively, with the remaining cases identified as indeterminate. A total of 811 (50.3%) female and 801 (49.7%) male deaths occurred. The median age at death was 65 years (Inter Quartile Range [IQR] = 49, 80 years). Most individuals who died were from rural (n = 1,312 [81.4%]), illiterate (n = 1,228 [76.2%]), and married (n = 928 [57.6%]). About 40% (n = 652) were farmers. Nearly all deaths occurred at home (n = 1,478 [91.7%]), with very few occurring in health institutions (n = 91, [5.6%]) (see Table 1).

**Table 1.** Socio-demographic characteristics of the deceased, Butajira HDSS, 2008–2019, Ethiopia.

Variable	Category	N (%)
Age	15–49	404(25.1)
	50–64	325(20.2)
	65+	883(54.8)
Sex	Female	811(50.3)
	Male	801(49.7)
Marital status	Married	928(57.6)
	Unmarried	159(9.9)
	Divorced	46(2.9)
	Separated	10(0.6)
	Widowed	469(26.1)
Education	Never been to school	1,228(76.2)
	Primary education	302(18.7)
	Secondary education	55(3.4)
	Higher education	27(1.7)
Occupation	Housewife	484(30)
	Farmer	652(40.4)
	Personal business	206(12.8)
	Other	270(16.7)
Place of death	Health institution	91(5.6)
	Home	1,478(91.7)
	Other place	43(2.7)
Residence	Urban	300(18.6)
	Rural	1,312(81.4)
	**Total**	**1,612(100)**

The distribution of deceased persons was further analysed by age and gender. Out of 1,612 adult deaths, 404 (25.1%) were young adults (15–49 years), 325 (20.2%) were middle-aged (50–64 years), and 883 (54.8%) were old-aged (65+ years). The sex-disaggregated proportion of adult deaths indicated that 28.6%, 19.2%, and 52.2% of male deaths were in the age group 15–49, 50–64, and 65+, respectively. On the other hand, 21.6%, 21.1%, and 57.3% of female deaths were in the age group 15–49, 50–64, and 65+, respectively (see Table 2).

**Table 2.** The distribution of deaths by adult age and gender, Butajira HDSS, 2008–2019, Ethiopia.

	Male (N = 801)						Female (N = 811)					
	15–49 (N = 229)		50–64 (N = 154)		65+ (N = 418)		15–49 (N = 175)		50–64 (N = 171)		65+ (N = 465)	
Year of death	N	%	N	%	N	%	N	%	N	%	N	%
2008	23	31.9	13	18.1	36	50	21	30.9	13	19.1	34	50
2009	19	28.8	13	19.7	34	51.5	17	29.8	12	21.1	28	49.1
2010	20	33.3	11	18.3	29	48.3	16	23.5	13	19.1	39	57.4
2011	14	37.8	5	13.5	18	48.6	17	25.8	15	22.7	34	51.5
2012	15	23.1	16	24.6	34	52.3	16	23.2	16	23.2	37	53.6
2013	29	43.9	13	19.7	24	36.4	18	25	23	31.9	31	43.1
2014	18	30	12	20	30	50	10	15.6	17	26.6	37	57.8
2015	18	23.4	22	28.6	37	48.1	13	23.2	3	5.36	40	71.4
2016	19	27.9	14	20.6	35	51.5	13	17.6	20	27	41	55.4
2017	22	25.9	14	16.5	49	57.6	15	17.2	19	21.8	53	60.9
2018	20	23.3	13	15.1	53	61.6	10	15.6	12	18.8	42	65.6
2019	12	20.3	8	13.6	39	66.1	9	13.6	8	12.1	49	74.2

### Broad causes of deaths in proportion

**Figure 1.**
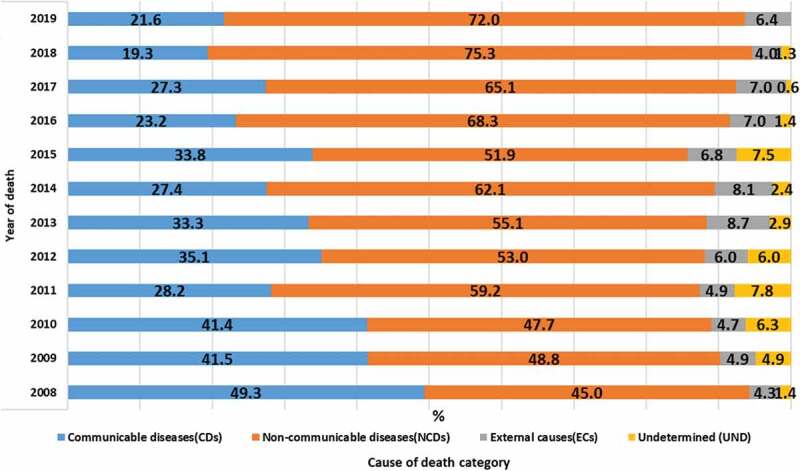
Temporal trend and pattern of broad CoD over the years(%), Butajira HDSS: 2008–2019, Ethiopia.

Broad CoD were grouped into CDs, NCDs, and ECs. Those with unspecified causes/ill-defined causes were categorised as undetermined. The number of deaths captured in each surveillance year, across the broad causes, is described in detail in Supplementary Table 2. Most proportional mortality was attributed to NCDs 950 (58.9%), followed by CDs 510 (31.1%), ECs 98 (6.0%), and undetermined causes of death 54 (3.3%). The trend in the proportional mortality due to CDs declined from 49.3% in 2008 to 21.6% in 2019, whereas the trend due to deaths from NCDs and ECs increased over the same period from 45.0% to 72.0% and 4.3% to 6.4%, respectively (see Figure 1).

The broad CoD were disaggregated by age, sex, and residence. The highest proportional mortality was attributable to NCDs and the lowest to ECs. The age pattern analysis indicated that 194 (48.0%), 199 (61.2%), and 557 (63.1%) deaths due to NCDs occurred among adults in the age group 15–49, 50–64, and 65+ years, respectively. Of NCD deaths, 450 (56.2%) and 500 (61.7%) were among males and females, while 765 (58.3%) and 185 (61.7%) were from rural residents and urbanites, respectively.

This study showed deaths due to CDs were comparably similar among males and females. NCD deaths were more common among females, and deaths due to ECs were more common among males. Generally, the proportional mortality due to CDs and ECs was higher among rural residents, whereas NCDs were higher in urban areas. The age differential analysis showed that CD deaths were more common among young adults (15–49). In contrast, NCD deaths were higher in both middle-age (50–64) and older adults (65+). Besides, deaths from EC were common in the age group 15–49 (see Table 3).

**Table 3.** Broad CoD distribution by sex, age, and residence, Butajira HDSS, 2008–2019, Ethiopia.

				Variable					
		Sex		Age			Residence		
		Female (N = 811)	Male (N = 801)	15–49 (N = 404)	50–64 (N = 325)	65+ (N = 883)	Rural (N = 1312)		Urban (N = 300)
YoD	CoD	% (95% CI)	%(95% CI)	%(95% CI)	%(95% CI)	%(95% CI)	%(95% CI)		%(95% CI)
2008	CDs	47.4 (35.7,59.1)	51.6(38.7,64.2)	63.6 (47.7,77.5)	50(22.9,70)	40(28.4,52.4)	51.3(41.8,60.6)		39.1(19.7,61.4)
	NCDs	50(38.3,61.6)	39.1(27.1,52)	29.5(16.7,45.2)	46.2(26.5,66.6)	54.3(41.9,66.2)	42.7(33.6,52.2)		56.5(34.4,76.8)
	ECs	2.6(0.3,9.1)	6.3(1.7,15.2)	6.8(1.4,18.6)	3.8(0,19.6)	2.9(0.3,9.9)	5.1(1.9,10.8)		-
	UND	-	3.1(0.3,10.8)	-	-	2.9(0.3,9.9)	0.9(0,4.6)		4.3(0.1,21.9)
2009	CDs	43.9(30.7, 57.6)	39.4(27.5,52.1)	55.6(38.3,72)	36(17.9,57.4)	35.5(23.7,48.6)	39.8(30,50.1)		48(27.7,68.6)
	NCDs	49.1(35.6,62.7)	48.5(35.9,61.1)	27.8(14.2,45.1)	52(31.3,72.2)	59.7(46.4,71.9)	49.0(38.7,59.2)		48(27.7,68.6)
	ECs	1.8(0.04,9.3)	7.6(2.5,16.8)	11.1(3.1,26)	8(0.9, 26)	-	5.1(1.6.11.5)		4(0.1,20.3)
	UND	5.3(1.1,14.6)	4.5(0.9,12.7)	5.6(0.6,18.6)	4(0.1,20.3)	4.8(1,13.4)	6.1(2.2,12.8)		-
2010	CDs	50.8(37.8,63.6)	32.3(21.2,45)	55.6(38.3,72)	33.3(15.6,55.3)	36.8(25.3,49.3)	41.7(32.1,51.8)		40(21.1,61.3)
	NCDs	39.7(27.5,52.7)	55.4(42.5,67.7)	30.6(16.3,48.1)	62.5(40.5,81.2)	51.5(39,63.7)	47.6(37.6,57.6)		48(27.7,68.6)
	ECs	1.6(0.0,8.5)	7.7(2.5,17)	11.1(3.1,26)	-	2.9(0.3,10.2)	4.9(1.5.10.9)		4(0.1,20.3)
	UND	7.9(2.6,17.5)	4.6(9,12.9)	2.8(0,14.5)	4.2(0.1,21.1)	8.8(3.3,18.2)	5.8(2.1,12.2)		8(0.9,26)
2011	CDs	35.2(22.6,49.3)	20.4(10.2,34.3)	31.0(15.2,50.8)	25(8.6,49.1)	27.8(16.4,41.6)	30(20.2,41.2)		21.7(7.4,43.7)
	NCDs	53.7(39.6,67.3)	65.3(50.3,78.3)	55.2(35.6,73.5)	70(45.7,88.1)	57.4(43.2,70.7)	55(43.4,66.1)		73.9(51.5,89.7)
	ECs	1.9(0.1,9.8)	8.2(2.2,19.6)	10.3(2.1,27.3)		3.7(0.4,12.7)	6.3(2,13.9)		-
	UND	9.3(3.1,20.3)	6.1(1.2,16.8)	3.4 (0,17.7)	5(0.1,24.8)	11.1(4.1,22.6)	8.8(3.5,17.2)		4.3(0.1,21.9)
2012	CDs	37.5(26.3,49.7)	32.3(20.9,45.3)	36.7(19.9,56.1)	43.8(26.3,62.3)	30.6(20.2,42.5)	36.6(26.8,47.1)		31.7(18,48)
	NCDs	52.8(40.6,64.6)	53.2(40.1,66)	50(31.2,68.7)	40.6(23.6,59.3)	59.7(47.4,71.1)	54.8(44.1,65.1)		48.8(32.8,64.8)
	ECs	2.8(0.3,9.6)	9.7(3.6,19.8)	10(2.1,26.5)	6.3(0.7,20.8)	4.2(0.8,11.6)	5.4(1.7,12.1)		7.3(1.5,19.9)
	UND	6.9(2.2,15.4)	4.8(1,13.4)	3.3(0,17.2)	9.4(1.9,25)	5.6(1.5,13.6)	3.2(0.6,9.1)		12.2(4,26,2)
2013	CDs	39.7(28,52.3)	27.1(17.1,39)	30.6(18.2,45.4)	32.4(17.3,50.5)	36.4(23.8,50.4)	34.7(25.4,4.7)		29.7(15.8,46.9)
	NCDs	52.9(40.4,65.1)	57.1(44.7,68.9)	51(36.3,65.5)	55.9(37.8,72.8)	58.2(44.1,71.3)	50.5(40.3,60.5)		67.6(50.2,81.9)
	ECs	4.4(0.9,12.3)	12.9(6,23)	12.2(4.6,24.7)	11.8(7.3,27.4)	3.6(0.4,12.5)	11.9(6.2,19.8)		-
	UND	2.9(0.3.10.2	2.9(0.3,9.9)	6.1(1.2,16.8)	-	1.8(0,9.7)	3.0(0.6,8.4)		2.7(0,14.1)
2014	CDs	23.8(13.9,36.2)	31.1(19.9,42,2)	23.3(9.9,42.2)	24.1(10.2,43.5)	30.8(19.9,43.4)	29.8(20.7,40)		20(7.7,38.5)
	NCDs	69.8((56.9,80.7)	54.1(40.8,66.9)	56.7(37.4,74.5)	69(49.1,84.7)	61.5(48.6,73.3)	58.5(47.8,68.5)		73.3(54.1,87.7)
	ECs	4.8(09,13.2)	11.5(4.7,22.2)	20(7.7,38.5)	6.9(0.8,22.7)	3.1(0.3,10.6)	9.6(4.4,17.3)		3.3(0,17.2)
	UND	1.6(0,8.5)	3.3(0.3,11.3)	-	-	4.6(0.9, 12.9)	2.1(0.2,7.4)		3.3(0,17.2)
2015	CDs	25.4(14.9,38.4)	40.5(29.2,52.5)	17.2(5.8,35.7)	41.7(22.1,63.3)	37.5(26.9,49)	38.5(28.7,49)		21.6(9.8,38.2)
	NCDs	61(47.4,73.4)	44.6(33,56.6)	62.1(42.2,79.3)	54.2(32.8,74.4)	47.5(36.2,58.9)	47.9(37.6,58.3)		62.2(44.7,77.5)
	ECs	6.8(1.8,16.4)	6.8(2.3,15)	6.9(0.8,22.7)	4.2(0.1,21.1)	7.5(2.8,15.6)	8.3(3.6,15.7)		2.7(0,14.1)
	UND	6.8(1.8,16.4)	8.1(3,16.8)	13.8(3.6,31.6)	-	7.5(2.8,15.6)	5.2(1.7,11.7)		13.5(4.5,28.7)
2016	CDs	22.2(12.7,34.4)	24.1(15.1,34.9)	19.4(7.4,37.4)	13.9(4.6,29.4)	29.3(19.3,40.9)	24.5(16.8,33.6)		18.8(7.2,36.4)
	NCDs	76.2(63.7,86)	62(50.4,72.7)	61.3(42.1,78.1)	77.8(60.8,89.8)	66.7(54.8,77.1)	68.2(58.6,76.7)		68.8(49.9,83.8)
	ECs	1.6(0,8.5)	11.4(5.3,20.5)	19.4(7.4,37.4)	5.6(0.6,18.6)	2.7(0.3,9.3)	6.4(2.5,12.6)		9.4(1.9,25)
	UND	-	2.5(3,8.8)	-	2.8(0,14.5)	1.3(0.7.2)	0.9(0,4.9)		3.1(0,16.2)
2017	CDs	28.7(19.8,38.9)	25.6(16.4,36.7)	20.5(9.2, 36.4)	30.3(15.5,48.7)	29(20.3,38.9)	26(19.1,33.7)		36.4(17.1,59.3)
	NCDs	67(56.5,76.3)	62.8(51.1,73.5)	48.7(32.4,65.2)	66.7(48.1,82)	71(61,79.6)	65.3(57.1,72.9)		63.6(40.6,82.8)
	ECs	3.2(0.6,9)	11.5 (5.4,20.7)	28.2(15,44.8)	3(0,15.7)	-	8(4.2,13.5)		-
	UND	1.1(0,5.7)	-	2.6(0,13.4)	-	-	0.7(0,3.6)		-
2018	CDs	14.3(7.3,24.1)	24.7(15.3,36.1)	17.2(5.8,35.7)	19.2(6.5,39.3)	20(12.4,29.4)	19.6(13.5,26.9)		-
	NCDs	83.1(72.8,90.6)	67.1(55.1,77.6)	69(49.1,84.7)	69.2(48.2,85.6)	78.9(69.3,86.6)	75(67.2,81.7)		100(15.8,1*)
	ECs	2.6(0.3,9)	5.5(1.5,13.4)	10.3(2.1,27.3)	11.5(2.4,30.1)	-	4.1(1.5,8.6)		-
	UND	-	2.7(0.3,9.5)	3.4(0,17.7)	-	1.1(0.5.7)	1.4(0.1,4.7)		-
2019	CDs	20(11.1,31.7)	23.3(13.3,36)	22.7(7.8,45.3)	25(7.2,52.3)	20.7(12.7,30.7)	22.1(15.1,30.5)		-
	NCDs	78.5(66.5,87.6)	65(51.5,76.8)	50(28.2,71.7)	75(47.6,92.7)	77(66.7,85.3)	71.3(62.4,79.1)		100(29.2,1*)
	ECs	1.5(0,8.2)	11.7(4.8,22.5)	27.3(10.7,50.2)	-	2.3(0.2,8)	6.6(2.8,12.5)		-
	UND	-	-	-	-	-			-

YoD: Year of Death, CoD: Causes of Death, (*) one-sided, 97.5% confidence interval.

### Mortality rate in broad causes of death

**Figure 2.**
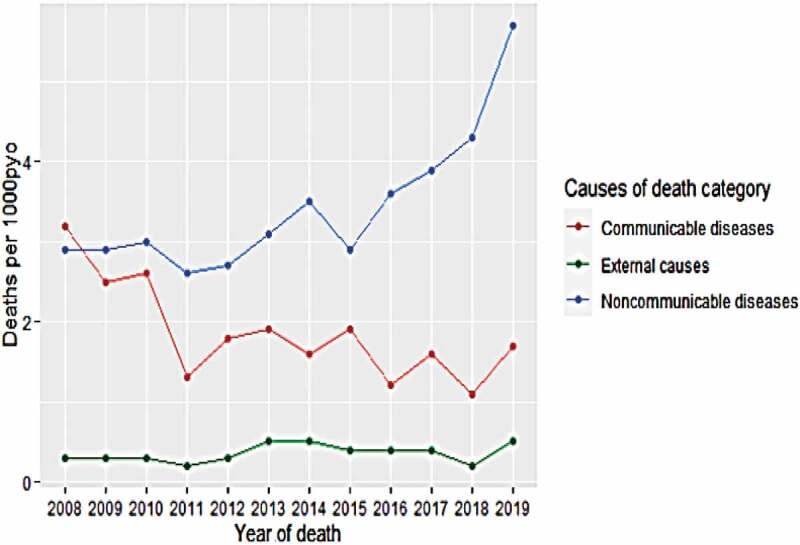
Trends in crude mortality rates (per 1000 person-years observed, pyo) by broad CoD in Butajira HDSS, 2008–2019, Ethiopia.

The overall adult mortality rate was 5.8 deaths per 1,000 person-years for the entire follow-up period between January 2008 and December 2019. The age-specific mortality rates in the age groups 15–49 years, 50–64 years, and 65+ were 1.7, 16.1, and 52.5 deaths per 1,000 person-years, respectively. On the other hand, the sex-specific mortality rate was 5.9 per 1,000 person-years among males and 5.8 per 1,000 person-years among females. Moreover, the mortality rate by residence type showed 3.4 deaths per 1000 person-years among urban residents and 6.8 deaths per 1000 person-years among rural counterparts (see Table 4). The trend analysis indicated that the adult mortality rate (per 1,000 person-years) due to NCDs and ECs was increasing. In contrast, a declining trend was observed due to CD deaths during the follow-up period (see Figure 2).

**Table 4.** Rate of adult mortality in broad causes of death during 2008–2019 in Butajira HDSS, Ethiopia.

				Variable						
				Age			Sex		Residence	
		Year of death	Rate/1000py (95%CI)	15–49 (95%CI)	50–64 (95%CI)	65+ (95%CI)	Male (95%CI)	Female	Urban	Rural
Causes of death category	CDs	2008	3.2(2.4,4)	1.5(0.9,2.1)	5.7(3,9.7)	33.0(22,47.3)	3.0(2,4.2)	3.4(2.3,4.7)	1.7(0.7,3.2)	3.7(2.8,4.7)
		2009	2.5(1.8,3.2)	1.1(0.6,1.6)	4.7(2.1,8.9)	25.9(16.3,38.9)	2.7(1.7,3.9)	2.3(1.4,3.3)	2.0(1,3.4)	2.7(1.9,3.6)
		2010	2.6(1.9,3.4)	1.1(0.6,1.6)	6.9(2.9,13.5)	26.4(17.1,38.7)	2.1(1.3,3.2)	3.2(2.1,4.5)	1.5(0.7,2.7)	3.2(2.3,4.3)
		2011	1.3(0.8,1.7)	0.4(0.1,0.7)	3.2(1,7.4)	13.4(7.5,22)	0.9(0.4,1.6)	1.5(0.9,2.3)	0.7(0.2,1.6)	1.5(0.9,2.2)
		2012	1.8(1.2,2.3)	0.5(0.2,0.8)	8.4(4.6,14)	17.5(11,26.3)	1.8(1,2.7)	1.7(1.1,2.4)	1.6(0.8,2.7)	1.8(1.2,2.5)
		2013	1.9(1.2,2.2)	0.7(0.3,1.1)	7.3(3.6,13)	16.2(9.9,24.9)	1.7(1,2.6)	2.0(1.3,2.9)	1.4(0.6,2.5)	2.1(1.4,2.9)
		2014	1.6(1,2.1)	0.4(0.1,0.8)	3.9(1.5,8)	11.8(7.2,18.1)	1.6(0.9,2.4)	1.5(0.8,2.4)	0.8(0.2,1.7)	1.9(1.2,2.7)
		2015	1.9(1.3,2.5)	0.2(0,0.4)	5.9(2.8,10.8)	16.3(11,23.1)	2.4(1.6,3.4)	1.3(0.7,2.1)	1.00.4,1.9)	2.3(1.6,3.1)
		2016	1.2(0.8,1.7)	0.3(0.1,0.6)	3.0(0.9,6.9)	10.7(6.7,16.1)	1.3(0.7,2)	1.1(0.6,1.8)	0.6(0.2,1.3)	1.5(0.9,2.1)
		2017	1.6(1.2,2.1)	0.3(0.1,0.5)	5.5(2.6,10)	12.6(8.4,18)	1.4(0.8,2.1)	1.9(1.2,2.7)	0.7(0.3,1.3)	2.3(1.6,3.1)
		2018	1.1(0.7,1.6)	0.2(0,0.4)	2.1(0.6,4.8)	8.0(4.8,12.4)	1.4(0.8,2.2)	0.8(0.3,1.4)	-	1.4(0.9,2)
		2019	1.7(1.1,2.4)	0.4(0.1,0.9)	3.2(0.8,8.1)	10.7(6.3,16.8)	1.7(0.9,2.8)	1.7(0.9,2.9)	-	2.3(1.5,3.3)
	NCDs	2008	2.9(2.2,3.7)	0.7(0.3,1.1)	5.2(2.6,9)	44.8(31.9,60.9)	2.3(1.4,3.3)	3.6(2.5,4.9)	2.5(1.3,4.2)	3.1(2.3,4)
		2009	2.9(2.2,3.7)	0.6(0.2,1.1)	6.8(2.8,10.2)	43.6(30.9,59.6)	3.4(2.3,4.7)	2.5(1.6,3.6)	2.0(1,3.4)	3.3(2.4,4.3)
		2010	3.0(2.3,3.8)	0.6(0.2,1)	13.0(6,19.2)	36.9(25.8,50.9)	3.6(2.5,4.9)	2.5(1.6,3.6)	1.8(0.9,3.1)	3.7(2.4,4.4)
		2011	2.6(2,3.3)	0.8(0.4,1.2)	9.1(4.9,15.2)	27.6(18.8,38.9)	3.0(2,4.2)	2.4(1.6,3.4)	2.4(1.3,3.8)	2.8(2,3.7)
		2012	2.7(2, 3.3)	0.6(0.3,0.9)	7.8(4.1,13.3)	34.3(24.9,45.9)	3.0(2,4.2)	2.4(1.6,3.2)	2.5(1.5,3.8)	2.7(2,3.5)
		2013	3.1(2.4,3.8)	1.1(0.7,1.6)	12.7(7.6,19.7)	25.9(17.7,36.3)	3.6(2.5,4.8)	2.7(1.8,3.7)	3.3(2.1,4.8)	3.0(2.2,3.9)
		2014	3.5(2.7,4.4)	0.9(0.5,1.4)	11.1(6.7,17)	23.7(16.9,32.1)	2.9(1.9,4)	4.3(3.2,5.7)	3.0(1.8,4.5)	3.8(2.8,4.9)
		2015	2.9(2.2,3.6)	0.9(0.5,1.4)	7.6(4,12.9)	20.7(14.6,28.3)	2.6(1.7,3.6)	3.2(2.2,4.4)	3.0(1.9,4.4)	2.9(2.1,3.8)
		2016	3.6(2.8,4.3)	0.8(0.4,1.2)	16.6(11,23.9)	24.3(18,31.9)	3.4(2.5,4.4)	3.8(2.8,5)	2.3(1.4,3.4)	4.2(3.3,5.2)
		2017	3.9(3.2,4.7)	0.8(0.4,1.2)	12.1(7.5,18.2)	30.8(24.1,38.6)	3.4(2.5,4.4)	4.4(3.3,5.6)	1.2(0.6,2)	5.7(4.6,6.9)
		2018	4.3(3.5,5.2)	0.9(0.5,1.3)	7.4(4.3,11.6)	31.7(25,39.5)	3.8(2.5,5)	4.9(3.7,6.2)	0.4(0,1.4)	5.3(4.3,6.3)
		2019	5.7(4.5,7)	0.9(0.4,1.6)	9.5(4.9,16.5)	39.7(30.9,50.1)	4.9(3.4,6.6)	6.7(4.9,8.8)	0.8(0.1,2.3)	7.4(5.9,9.1)
	ECs	2008	0.3(0.1,0.6)	0.2(0,0.5)	0.4(0,2.2)	2.4(0.2,8.6)	0.4(0.1,1)	0.2(0,0.7)	-	0.4(0.1,0.8)
		2009	0.3(0.1,0.6)	0.2(0,0.5)	1.0(0.1,3.6)	-	0.5(0.1,1.1)	0.1(0,0.5)	0.2(0,1.1)	0.3(0,0.7)
		2010	0.3(0.1,0.6)	0.2(0,0.5)	-	2.1(0.2,7.5)	0.5(0.1,1.1)	0.1(0,0.5)	0.1(0,1.1)	0.4(0.1,0.9)
		2011	0.2(0,0.5)	0.1(0,0.2)	-	1.8(0.2,6.4)	0.4(0.1,1)	0.1(0,0.5)	-	0.3(0,0.7)
		2012	0.3(0.1,0.5)	0.1(0,0.2)	1.2(0.1,4.3)	2.4(0.4,6.9)	0.5(0.1,1.1)	0.1(0,0.3)	0.4(0,1.1)	0.3(0,0.7)
		2013	0.5(0.2,0.8)	0.3(0.1,0.6)	2.7(0.7,6.9)	1.6(0.1,5.7)	0.8(0.3,1.5)	0.2(0,0.5)	-	0.7(0.3,1.2)
		2014	0.5(0.2,0.8)	0.3(0.1,0.6)	1.1(0.1,3.9)	1.2(0.1,4.3)	0.6(0.2,1.2)	0.3(0,0.8)	0.1(0,1.1)	0.6(0.2,1.1)
		2015	0.4(0.1,0.7)	0.1(0,0.3)	0.6(0,3.3)	3.3(1.2,7.1)	0.4(0.1,0.9)	0.4(0.1,1)	0.1(0,1.1)	0.5(0.2,0.9)
		2016	0.4(0.1,0.6)	0.3(0.1,0.6)	1.2(0.1,4.3)	1.0(0.1,3.6)	0.6(0.2,1.1)	0.1(0,0.5)	0.3(0,0.8)	0.4(0.1,0.8)
		2017	0.4(0.2,0.7)	0.5(0.2,0.8)	0.5(0,2.7)	-	0.6(0.2,1.1)	0.2(0,0.5)	-	0.7(0.3,1.2)
		2018	0.2(0,0.5)	0.1(0,0.2)	1.2(0.2,3.5)	-	0.3(0,0.7)	0.2(0,0.7)	-	0.3(0.1,0.6)
		2019	0.5(0.2,1)	0.5(0.2,1.3)	-	1.2(0.1,4.3)	0.9(0.3,1.8)	0.1(0,0.5)	-	0.7(0.3,1.3)

YoD: Year of Death, CoD: Causes of Death.

An overall reduction in the adult mortality rate from 3.2 in 2008 to 1.7 in 2019 due to CDs indicates a declining trend. A significant downward trend in CD deaths was observed in the trend analysis test statistics (Mann–Kendall trend test, with *tau* = −0.523 and *P* < 0.05) with an overall adult mortality rate of 1.9 deaths per 1,000 person-years. The age-specific mortality rate per 1,000 person-years due to CDs declined from 1.5 to 0.4, 5.7 to 3.2, and 33.0 to 10.7 between 2008 and 2019 in the age groups 15–49, 50–64, and 65+, respectively. On average, age-specific mortality rates of 0.6, 4.9, and 16.9 in CDs per 1,000 person-years were observed in the age groups 15–49, 50–64, and 65+, respectively.

The adult mortality rates due to NCDs increased from 2.9 in 2008 to 5.7 in 2019 deaths per 1,000 person-years. The 12-year trend test in NCD death (*tau* = 0.698, *P* < 0.05) indicated a significant upward trend in the cause-specific mortality rate, with an overall adult mortality rate of 3.4 deaths per 1,000 person-years. Change in the adult mortality rate per 1,000 person-years due to NCDs was observed from 0.7 to 0.9, 5.2 to 9.5, and 44.8 to 39.7 between 2008 and 2019 in the age groups 15–49, 50–64, and 65+, respectively. On average, the mortality rates in NCDs were 0.8, 9.9, and 32.0 deaths per 1,000 person-years in the age groups 15–49, 50–64, and 65+, respectively.

An increasing trend in adult mortality rates from 0.3 deaths per 1,000 person-years in 2008 to 0.5 in 2019 due to ECs was obtained with an overall adult mortality rate of 0.4 deaths per 1,000 person-years. The trend analysis (*tau* = 0.287, *P* > 0.05) indicated an increasing but non-significant trend in death due to ECs. The adult mortality rate due to ECs per 1,000 person-years changed from 0.2 to 0.5, 0.4 to 0.0, and 0.4 to 0.78 between 2008 and 2019 in the age groups 15–49, 50–64, and 65+, respectively. On average, ECs death rates of 0.2, 0.8, and 1.4 per 1,000 person-years in the age groups 15–49, 50–64, and 65+, respectively, indicate an upward trend.

**Figure 3.**
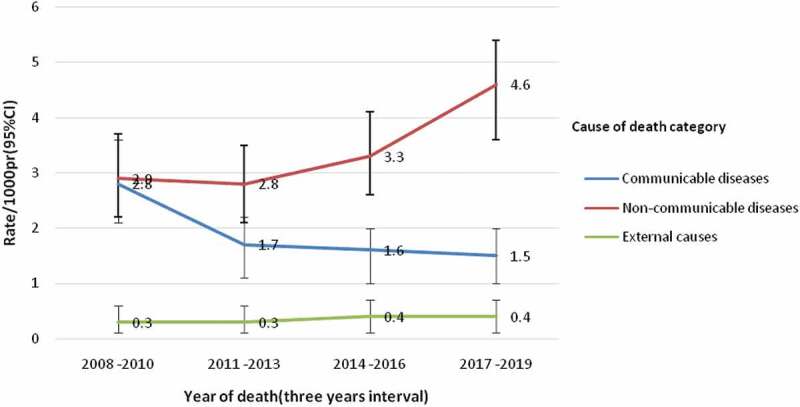
The 3-years moving average in the adult mortality rate during 2008–2019 in Butajira HDSS, Ethiopia.

In addition, the 3-year moving averages of adult mortality rates per 1,000 person-years from 2008 to 2019 (2009–2010, 2011–2013, 2014–2016, and 2017–2019) showed an increasing trend in mortality due to NCDs and ECs, whereas a downward trend was observed in the CD deaths (see Figure 3).

Considering all deaths, males had a marginally higher mortality rate than females (5.9:5.8). Moreover, the average mortality rate in CDs was similar among males and females (1.8:1.9), but it increased marginally for females in NCDs (3.6:3.3) and was significantly higher among males in ECs (1.5:0.12). Moreover, in rural areas, death rates were more pronounced in all broad CoD categories.

## Discussion

This study highlights pertinent temporal trends in community-level CoD in Ethiopia. Change in the adult mortality rate from 3.2 (95% CI: 2.4,4.0) to 1.7 (95% CI: 1.1,2.4), 2.9 (95% CI: 2.2,3.7) to 5.7 (95% CI: 4.5,7.0), and 0.3 (95% CI: 0.1,0.6) to 0.5 (95% CI: 0.2,1.0) between 2008 and 2019 due to CDs, NCDs, and ECs, respectively, suggests an increasing trend of adult mortality rate in NCDs and ECs and a declining trend in CDs. This VA-based study in Ethiopia showed a declining mortality trend from 43.3 in 2007 to 12.3 in 2017 due to CDs, maternal conditions, and nutritional deficiencies [40].

This study identified the crude adult mortality rate of 5.8 (95%CI: 5.2,5.8) per 1,000 person-years. It is lower than 7.8 (95%CI: 7.4,8.0) per 1,000 person-years reported from Butajira HDSS [41]. This variation can be explained by the difference in surveillance years, the age range used for the analysis, and the data source. The former study used mortality information available in the demographic database between the years 1987 and 2004 for the age group 15–64. It is also lower than the 10.4 per 1,000 person-years of adult mortality rate reported in Ghana [42]. Difference in the duration and year of surveillance (2006–2010), the VA interpretation method, population characteristics, and study setting are possible reasons for this difference. Furthermore, the health extension programme initiative and the transitioning of essential health services in Ethiopia have contributed to this mortality decline [43].

As age increases, the overall mortality rate increases, a similar finding was reported from Kersa-HDSS, Ethiopia, where more deaths were observed among older adults of 65 years and above [44]. However, considering all deaths, males had a marginally higher mortality rate than females. As age increases, the adult mortality rate (per 1,000 population) among men is higher [44–46]. The higher adult mortality rate per 1,000 person-years in rural areas in this study is consistent with findings from Kersa-HDSS in Ethiopia [44]. This study also identified an average adult mortality rate of 1.9, 3.4, and 0.4 in CDs, NCDs, and ECs, respectively. Comparably, the Kombewa-HDSS site in Kenya found a death rate of 2.6 from CDs and 2.1 from NCDs per 1,000 person-years, but CDs were the leading CoD [47]. Moreover, the proportional mortality of 31.1% in CDs, 58.9% in NCDs, and 6% in ECs in this study differs from the 2018 WHO report of 49%, 39%, and 12%, respectively [48]. This variation is likely to be due to the difference in the study design and data collection methods.

This study also identified an increasing trend in the NCD death rates, similar to other studies conducted in Ethiopia [40,49–51]. NCDs are a growing health burden in Africa [25,52,53] and 77% of all NCD deaths in LMICs [54]. Increasing exposure to modifiable risk factors for NCDs, including tobacco use, physical inactivity, harmful use of alcohol, and unhealthy diets [43], is one explanation. Similarly, a substantial increase in NCDs risk exposure is evident within the Ethiopian community [50,55]. Despite these trends, routine clinical practices and the health care system in Ethiopia are focused on CDs [56].

Considering the age–sex dimensions, the NCD death rate increased, as age increased. Similar findings have been reported from Dabat-HDSS, Ethiopia, where the 50+ age group has a higher risk of death due to NCDs [57]. Furthermore, a VA-based study in South Africa [58] and Tanzania [59] showed NCD deaths increased with age and were the major CoD in the 65 and over age group. Moreover, NCD deaths were marginally higher among women, but higher mortality rate of NCDs among men was reported in a previous study from Butajira HDSS [60,61]. Sex differences in mortality associated with social, behavioural, and biological factors have been reported elsewhere [62]. A relatively higher mortality rate was identified in rural areas. This result is similar to that of the research conducted in Kersa and Butajira HDSS [44,61]. The increased exposure to NCDs’ behavioural risk factors among rural dwellers is a possible reason [63].

The CD deaths reported in this study were comparable with research reports in HDSS-based studies from Kilite Awlaelo (34.9%) [51] and Kersa (32.4%) in Ethiopia [44] but differ from a study in Dabat-HDSS, Ethiopia (48.0%) [57]. This variation may be due to variability in the surveillance years and the studied population. Differences have been observed in the institutional set-up, geographical locations [64,65], and methodology among different HDSS sites [65]. Butajira HDSS is a historic site with long-standing experience in community awareness about CDs in the catchment area, and this might contribute to the reduction of CD deaths.

The 1998 burden of disease study in the Butajira HDSS showed that 82.4% of deaths were due to communicable, maternal, and prenatal conditions [66]. In Butajira-HDSS, from 1995 to 1999, CDs accounted for 60.8% of deaths [60] and from 1987 to 2004, 53% of deaths were attributed to CDs [61]. A remarkable decline in mortality has occurred for major CDs in Ethiopia [67]; however, challenges persist [43,49]. This downward trend is a probable indication of the country’s effort to reduce CD-related deaths. The scaling up of health extension programmes and recent efforts to end major CDs by 2030 [43] may have played a significant role.

The proportion of adult mortality due to CDs was higher in the elderly (65+). Although it has been linked to physiologic changes and a decline in immune function [56], CDs have a significant impact on young adults [57,58,68,69]. CD deaths are comparable between both sexes. Similarly, CD mortality did not differ significantly by gender [61]. This study further identified that the adult mortality rate in CDs is almost double in rural compared with urban areas. In the rural areas, basic life and health needs are not adequately available and communities are susceptible to infectious diseases [44].

In this study, a considerable proportion of deaths were attributed to ECs. Mortality caused by injuries is becoming a global public health concern [70]. Death from ECs in Ethiopia varies in studies, for example, 6.0% in a verbal autopsy study in Addis Ababa [71], 6.4% in pooled data from five HDSS sites in Ethiopia [27], 10.4% in Dabat-HDSS [57], and 12.3% in Kilite Awlaelo-HDSS [51]. However, the current finding is higher than in the previous Butajira-HDSS study (0.9%) [60]. Increased mortality from injuries may be associated with poor infrastructure, especially in deaths due to transportation accidents. The variation might also be due to substantial community-level differences among HDSS in Ethiopia [72]. High rugged mountains in the northern part of the Ethiopian contribute to deaths due to both man-made and natural causes.

The proportion of adult mortality due to ECs was higher in young adults (15–49). In agreement with the current study, deaths were higher among people aged 15–34, with observable variation across ages [50]. In contrast, older individuals with a higher risk of death due to injuries were reported from Rufiji HDSS in Tanzania [73]. Another study also described that age variation in ECs death is linked with specific causes [74]. The current study further identified higher ECs deaths among men than women. Similarly, other studies indicated that males had a higher risk of death due to accidents than females [51,73,75].

The complex interplay among demographic, epidemiological, and risk factors influences the changing patterns and trends in mortality. Socio-economic development and progress in reducing CDs have led to a growing burden of NCDs in LMICs [76,77]. The burden shift revealed in this study suggests a nationwide epidemiological transition. The growing mortality trend in NCDs can be explained by changes in lifestyle, dietary habits, and urbanisation [43,78]. Despite variation in the rates, the epidemiological transition is occurring in most African countries [25]. As in other developing countries, Ethiopia is currently facing a triple burden due to CDs, NCDs, and ECs [43]. This situation is escalating pressure on the health care system.

## Strengths and limitations of the study

The main strength of this study is the ability to get consistent, timely, and empirical CoD data at the population level from a historical cohort, the Butajira-HDSS. Yet there are some limitations associated with the reliability and validity of VA CoD assigning and interpretation procedures, in particular, for diseases having less specific clinical pictures. The inter- and intra-observer differences in the PCVA method and inflexibility in determining the CoD by the CCVA method can affect the VA’s precision compared to the true gold standard for CoD determination. Repetition of individual ID numbers and other coding issues were solved via intensive data cleaning before this study’s commencement. Very few cases (<1%) with data inconsistency were dropped from the current analysis.

## Conclusion

The current VA-based study highlights deaths due to CDs, NCDs, and ECs as an ongoing public health burden with variation across age-gender. Despite Ethiopia’s remarkable progress in improving the health sector, CDs, NCDs, and ECs present a triple burden. Pragmatically, this situation presents challenges in this era of Sustainable Development Goals. Public health policies should accommodate feasible integrated control strategies for CDs and NCDs within context-specific and suitable health system platforms. Moreover, public awareness about NCDs risk behaviours and habits like tobacco and harmful alcohol use, unhealthy diets, and physical inactivity should be advocated to avert premature mortality.

## Supplementary Material

Supplemental Material

## Paper context

The paucity of information on deaths and their causes critically impedes the complete understanding of the problem. A verbal autopsy is a well-established method that provides information on the level, trend, and pattern of deaths, especially in low-income settings. In Ethiopia, deaths from non-communicable diseases and external causes are rising but declining for communicable diseases. The epidemiological transition in poorer countries is escalating health care system stress. Context-specific health system reforms are urgently needed.
