# Supplementary material for: The trend and pattern of adult mortality in South-Central Ethiopia: analysis using the 2008-2019 data from Butajira Health and Demographic Surveillance System
Source: Glob Health Action. 2022 Sep 30;15(1):2118180. doi: 10.1080/16549716.2022.2118180 (PMC9542780; doi:10.1080/16549716.2022.2118180)
Supplement: Supplemental Material [file ZGHA_A_2118180_SM4044.zip › Sup_table_2._Temp_trend_in_the_broad_CoD.doc]

Supplementary table 2. Temporal trend in the number of broad CoD

Causes of death category

| Year of death | Communicable diseases(CDs) | Non-communicable diseases(NCDs) | External causes(ECs) | Undetermined (UND) | Total |
| --- | --- | --- | --- | --- | --- |
| 2008 | 69 | 63 | 6 | 2 | 140 |
| 2009 | 51 | 60 | 6 | 6 | 123 |
| 2010 | 53 | 61 | 6 | 8 | 128 |
| 2011 | 29 | 61 | 5 | 8 | 103 |
| 2012 | 47 | 71 | 8 | 8 | 134 |
| 2013 | 46 | 76 | 12 | 4 | 138 |
| 2014 | 34 | 77 | 10 | 3 | 124 |
| 2015 | 45 | 69 | 9 | 10 | 133 |
| 2016 | 33 | 97 | 10 | 2 | 142 |
| 2017 | 47 | 112 | 12 | 1 | 172 |
| 2018 | 29 | 113 | 6 | 2 | 150 |
| 2019 | 27 | 90 | 8 |  | 125 |
| **Total** | **510** | **950** | **98** | **54** | 1612 |
