# Supplementary material for: The trend and pattern of adult mortality in South-Central Ethiopia: analysis using the 2008-2019 data from Butajira Health and Demographic Surveillance System
Source: Glob Health Action. 2022 Sep 30;15(1):2118180. doi: 10.1080/16549716.2022.2118180 (PMC9542780; doi:10.1080/16549716.2022.2118180)
Supplement: Supplemental Material [file ZGHA_A_2118180_SM4044.zip › Supplementary_Table_1._InterVA4_CoD_8.doc]

**Supplementary documents**

Supplementary Table 1. The interVA4 cause of death codes and its ICD-10 equivalent codes

| **InterVA4 cause of death** | **VA Cause of death (ICD-10 code)** |
| --- | --- |
| Sepsis (non-obstetric) | Infectious diseases, unspecified [A40-A41] |
| Acute resp infect incl pneumonia | Acute lower respiratory infections (including pneumonia and acute bronchitis) [J12–J18, J00–J11, J20–J22] |
| HIV/AIDS related death | HIV/AIDS [B20-B24] |
| Diarrhoeal diseases | Intestinal infectious diseases (including diarrhoeal diseases) [A00-A09] |
| Malaria | Malaria [B50-B54] |
| Measles | Measles [B05] |
| Meningitis and encephalitis | Meningitis [G009, G039, G049] |
| Tetanus | Infectious diseases, unspecified [A35] |
| Pulmonary tuberculosis | Tuberculosis [A15-19] |
| Pertussis | Infectious diseases, unspecified [A379] |
| Haemorrhagic fever | Other specified infectious and parasitic diseases [B889] |
| Other and unspecified infect dis | Infectious diseases, unspecified [B99] |
| Oral neoplasms | Malignant neoplasm of lip, oral cavity and pharynx [C002,C005,C009,C029,C039,C049,C059,C069,C089,C099,C109,C119,C139,C140] |
| Digestive neoplasms | Malignant neoplasms of digestive organs [C15-C26] |
| Respiratory neoplasms | Malignant neoplasm of trachea, bronchus and lung [C30-C39] |
| Breast neoplasms | Malignant neoplasm of breast [C50] |
| Reproductive neoplasms MF | Malignant neoplasms of male and female genital organs [C51-C58; C60-C63] |
| Other and unspecified neoplasms | Other and unspecified neoplasm [C07-C14; C40-C49; C60-D48] |
| Severe anaemia | Other and unspecified non communicable disease [R99] |
| Severe malnutrition | Lack of food and/or water [X53-X54] |
| Diabetes mellitus | Diabetes mellitus [E10-E14] |
| Acute cardiac disease | Ischaemic heart disease [I20-I25] |
| Sickle cell with crisis | Other and unspecified non communicable disease [R99] |
| Stroke | Stroke [I64] |
| Other and unspecified cardiac dis | Other and unspecified cardiac disease [I99] |
| Chronic obstructive pulmonary dis | Chronic obstructive lung disease [J40-J44] |
| Asthma | Asthma [J45-J46] |
| Acute abdomen | Acute abdomen [R10] |
| Liver cirrhosis | Chronic liver disease [K70-K76] |
| Renal failure | Renal failure [N17-N19] |
| Epilepsy | Epilepsy [G40-G41] |
| Other and unspecified NCD | Other and unspecified non communicable disease [D55-D89; E00-E07; E15-E35; E50-E90; F00-F99; G06-G09; GG10-G37; G50-G99; H00-H95; J30-J39; J47-J99;K00-K31; K35-K38; K40-K93; L00-L99; M00-M99; N00-N16; N20-N99; R00-R09; R11-R94] |
| Congenital malformation | Other specified disorders related to perinatal period [P022] |
| Road traffic accident | Pedestrian injured in traffic accident [V01-V89] |
| Other transport accident | Other transport accident [V90-V99] |
| Accid fall | Accidental fall [W00-W19] |
| Accid drowning and submersion | Accidental drowning and submersion [W65-W74] |
| Accid expos to smoke fire & flame | Accidental exposure to smoke, fire and flames [X00-X19] |
| Contact with venomous plant/animal | Contact with venomous animals and plants [X20-X29] |
| Exposure to force of nature | Exposure to force of nature [X30-X39] |
| Accid poisoning & noxious subs | Accidental poisoning and exposure to noxious substance [X40] |
| Intentional self-harm | Intentional self-harm [X60-X84] |
| Assault | Assault [X85-Y09] |
| Other and unspecified external CoD | Accident, unspecified [S00-T99; W20-W64; W75-W99; X50-X59; Y10-Y98] |
| Ectopic pregnancy | Ectopic pregnancy [O009] |
| Abortion-related death | Abortion [O019,O029,O033,O038,O053] |
| Pregnancy-induced hypertension | Hypertensive disorders of pregnancy [O10-O16] |
| Obstetric haemorrhage | Ante, intra and postpartum haemorrhage [O46; O67; O72] |
| Obstructed labour | Obstructed labour [O63-O66] |
| Pregnancy-related sepsis | Puerperal sepsis [O85-O86] |
| Anaemia of pregnancy | Anaemia of pregnancy [O99] |
| Ruptured uterus | Obstetric trauma [O71] |
| Other and unspecified maternal CoD | Other and unspecified maternal CoD [O01-O02; O20-O45; O47-O62; O68-O70; O73-O84; O86-O99] |
