# Supplementary material for: The trend and pattern of adult mortality in South-Central Ethiopia: analysis using the 2008-2019 data from Butajira Health and Demographic Surveillance System
Source: Glob Health Action. 2022 Sep 30;15(1):2118180. doi: 10.1080/16549716.2022.2118180 (PMC9542780; doi:10.1080/16549716.2022.2118180)
Supplement: Supplemental Material [file ZGHA_A_2118180_SM4044.zip › Supporting_document_Key_questions.doc]

**Key questions**

**What is already known?**

Reliable and timely data are critically important to indicate the levels, patterns, and trends of mortality over time. However, paucity of information on deaths critically impedes complete understanding of  the burden due to CDs, NCDs and ECs, especially in rural settings. VA-based cause of death study in the Health and Demographic Surveillance Systems (HDSS) provides data-driven and context-specific health policy inputs in areas where death registration systems are incomplete.

**What are the new findings?**

NCDs were the major cause of death, a considerable proportion of CDs death while extra burden associated with ECs. There is a significant declining trend of adult mortality rate in CDs but a significant parallel increase in NCDs death rate. The disaggregated results from this study further indicated that adult mortality levels and trends mainly varied across the age-gender dimensions.

**What do the new findings imply?**

A triple burden due to CDs, NCDs, and ECs could increase the risk of premature mortality and pose a huge health care system stress. The noticeable burden shift shown in NCDs and CDs implicates a clue to an epidemiological transition in the study area. In similar settings, further empirical research needs to be carried out to initiate nationwide health policies reforms and set up suitable health system platforms.
